# Radiosensitization of PC3 Prostate Cancer Cells by 5-Thiocyanato-2′-deoxyuridine

**DOI:** 10.3390/cancers14082035

**Published:** 2022-04-18

**Authors:** Magdalena Zdrowowicz, Magdalena Datta, Michał Rychłowski, Janusz Rak

**Affiliations:** 1 Laboratory of Biological Sensitizers, Faculty of Chemistry, University of Gdańsk, Wita Stwosza 63, 80-308 Gdańsk, Poland; magdalena.datta@phdstud.ug.edu.pl (M.D.); janusz.rak@ug.edu.pl (J.R.); 2 Laboratory of Virus Molecular Biology, Intercollegiate Faculty of Biotechnology of University of Gdańsk and Medical University of Gdańsk, Abrahama 58, 80-307 Gdańsk, Poland; michal.rychlowski@biotech.ug.edu.pl

**Keywords:** radiosensitizer, modified nucleosides, clonogenic assay, radiosensitivity

## Abstract

**Simple Summary:**

Radiation therapy is one of the main treatments for cancer. However, the success of treatment by radiation therapy is largely dependent on tumor radiosensitivity. To improve therapeutic outcomes, radiation therapy should be combined with the use of a radiosensitizer which enables irradiation at lower doses with higher efficacies. 5-Thiocyanato-2′-deoxyuridine has been reported as a potential radiosensitizer of DNA damage based on advanced radiation chemical studies. In this paper, for the first time, we demonstrate the radiosensitizing properties of this modified nucleoside at the cellular level. The tested analogue increases the sensitivity of prostate cancer cells to ionizing radiation which is, at least partially, related to an increase in the number of DNA double-strand breaks and cell cycle regulation.

**Abstract:**

Purpose: The radiosensitizing properties of uracil analogs modified in the C5 position are very interesting in the context of their effectiveness and safety in radiation therapy. Recently, radiation chemical studies have confirmed that 5-thiocyanato-2′-deoxyuridine (SCNdU) undergoes dissociation induced by an excess electron attachment and established this nucleoside as a potential radiosensitizer. In this paper, we verify the sensitizing properties of SCNdU at the cellular level and prove that it can effectively enhance ionizing radiation-induced cellular death. Methods and Materials: Prostate cancer cells were treated with SCNdU and irradiated with X rays. The cytotoxicity of SCNdU was determined by MTT test. Cell proliferation was assessed using a clonogenic assay. Cell cycle analyses, DNA damage, and cell death analyses were performed by flow cytometry. Results: SCNdU treatment significantly suppressed the proliferation and increased the radiosensitivity of prostate cancer cells. The radiosensitizing effect expressed by the dose enhancement factor is equal to 1.69. Simultaneous exposure of cells to SCNdU and radiation causes an increase in the fraction of the most radiosensitive G2/M phase, enhancement of the histone H2A.X phosphorylation level, and apoptosis induction. Finally, SCNdU turned out to be marginally cytotoxic in the absence of ionizing radiation. Conclusions: Our findings indicate that SCNdU treatment enhances the radiosensitivity of prostate cancer cells in a manner associated with the cell cycle regulation, double strand formation, and a slight induction of apoptosis.

## 1. Introduction

Cancer is the main cause of death in highly developed countries, while radiation therapy (RT) is a basic modality used to treat this disease [1]. Solid cancers (tumors) include 50% of all cases and are characterized by a high level of hypoxia, which makes them radioresistant [2]. The radioprotectant effect of hypoxia (healthy, oxygenated cells surrounding the tumor are more sensitive to ionizing radiation than those targeted by RT) and other radio-resistance factors can be reduced or even eliminated by introducing radiosensitizers, i.e., substances that sensitize cells to radiation [3,4,5]. Various radiosensitization strategies are also being developed, e.g., Okuyama et al. reported that HeLa cells could be synchronized in early M-phase after treatment with a novel anti-microtubule agent, KPU-300, consequently leading to significant synergistic decrease of surviving fraction after the subsequent radiation exposure [6]. Due to the fact that the number of radiosensitizers employed in clinics is surprisingly small, it seems rational to look for and test new potentially radiosensitizing molecules. One of the widely studied groups of sensitizers are uracil analogues such as 5-bromo-2′-deoxyuridine (BrdU) [7,8]. Their sensitization mechanism is likely related to the rapid reaction of the modified nucleobase with hydrated electrons which are one of the main products of the water radiolysis [9] that takes place during the exposure of cells to ionizing radiation and which are not utilized by classical RT. The primary anions formed as a result of electron attachment to BrdU undergo bromide anion elimination, yielding the highly reactive uridine-5-yl radical (dU^•^) [10,11]. If the latter species is generated in DNA, then a secondary hydrogen atom transfer from the adjacent sugar moiety can ultimately lead to a DNA strand break [12,13,14]. A representative of uracil derivatives with potential radiosensitizing properties is also 5-thiocyanato-2′-deoxyuridine (SCNdU) [15,16]. A combination of theoretical studies together with negative ion photoelectron spectroscopy experiments demonstrated that this analogue possesses a propensity to undergo dissociation due to an excess electron [15]. Further studies on the mechanism of the electron-induced degradation of SCNdU show that electron attachment to SCNdU leads to two parallel reactions producing quite different products [16]. The first one is related to the S-CN bond cleavage in the thiocyanate substituent which consequently leads to the formation of highly cytotoxic individuals: the cyanide anion (CN^−^) and the 5-thiouridyl radical (dU-5-S^•^). The latter stabilizes itself as a result of dimerization (dU-S-S-dU). The second path causes C5-S bond cleavage and a secondary, equally toxic dU^•^ is formed which is stabilized by the formation of 2′-deouxyuridine (dU) [16]. These radiation chemical studies established SCNdU as a potential radiosensitizer, but it has not yet been proven to be a sensitizing agent at the cellular level. Therefore, the aim of this paper was to verify the radiosensitizing properties of SCNdU in cancer cells. A human prostate cancer cell culture was used in the study because overcoming its radioresistance still remains a challenge for current RT. In this paper, we demonstrate, by a clonogenic assay, the cytotoxic activity of SCNdU and the enhancement of PC3 cell sensitivity to radiation after treatment with the tested compound. To clarify the mechanism of radiosensitization, we used flow cytometry to examine the effects of combined treatment (SCNdU and ionizing radiation) on cell cycle regulation, histone H2A.X phosphorylation (molecular marker of double strand breaks), and cell death.

## 2. Materials and Methods

### 2.1. Cell Culture

PC3 cells were grown in F12k medium supplemented with 10% FBS and antibiotics at a concentration of 100 U/mL. The cell lines were obtained from ATCC and maintained at 37 °C in a humidified atmosphere with 5% CO_2_. The plates with cells were irradiated using a CellRad X-ray cabinet (Faxitron, X-ray Corporation, Tucson, AZ, USA). SCNdU was resuspended in ultrapure water for treatment in all cellular experiments performed.

### 2.2. Synthesis of SCNdU

The derivative was synthesized and purified according to the method described in the literature, which is based on the formation of ClSCN in anhydrous acetic acid solution, into which 2′-deoxyuridine was introduced [15]. The chromatogram of SCNdU after synthesis and HPLC purification is depicted in the Supplementary Materials (Figure S1).

### 2.3. Cytotoxicity Assay

MTT test was used to determine cytotoxicity. Cells were seeded in 96-well plates at a density of 4 × 10^3^ per well and incubated (37 °C, 5% CO_2_) overnight. Then, the cells were treated with SCNdU in the concentration range of 0.01 to 100 µM or with the same volume of the vehicle (H_2_O) alone as a control. The prepared plates were further incubated for 24, 48, and 72 h. Then, aqueous MTT salt solutions (4 mg/mL, 25 µL/well) were added. After 4 h of incubation, the formazan product was dissolved in DMSO. The absorbance was measured at 570 nm (660 nm was used as reference wavelength) using an EnSpire microplate reader (Perkin Elemer, Waltham, MA, USA). The control cell viability was assumed to be 100%. Three independent experiments were carried out in three repetitions.

### 2.4. Cell Cycle Analysis

PC3 cells were divided into four groups: the control group (H_2_O), the group treated with SCNdU (50 μM), the irradiated (5 Gy, Cellrad X-ray cabinet, Faxitron X-ray Corporation, 4.14 Gy × min^−1^) group, and the combination group (exposed to 50 μM SCNdU and irradiated with 5 Gy). The time of incubation with SCNdU was 24 h. The concentration of SCNdU was optimized on the basis of preliminary studies taking into account the effectiveness and toxicity of the tested analog. In an experiment showing only the effect of SCNdU (without irradiation) on the distribution of cell cycle phases, the cells were incubated for 48 h with the test compound in the 0–100 µM concentration range (see Figure S2 in the Supplementary Material).

Flow cytometry: The cells were dissociated with accutase solution 24h after irradiation, fixed by ice-cold 70% ethanol, and stained with propidium iodide (Guava Cell Cycle Reagent, Luminex, Austin, TX, USA) for 30 min, according to the manufacturer’s protocol. Then the probes were analyzed by flow cytometry (Guava easyCyte 12, Merck Millipore, Darmstadt, Germany). The cells were gated on the scatter plot FSC (Forward Scatter) versus DNA content of the cell sample for exclusion of cell debris and cell aggregates. The cells in different phases of the cell cycle were evaluated by the help of Incyte 3.3 software.

### 2.5. Clonogenic Assay

The PC3 cells were grown in the presence of SCNdU (50 µM) or vehicle (H_2_O) as a control for 24 h (2 × 10^5^ cells, 37 °C and 5% CO_2_, and F12k medium supplemented with antibiotics and 10% bovine serum). After 24 h, they were exposed to the indicated doses of ionizing radiation: 0, 0.5, 1, 2, 3, and 4 Gy. After a further 6 h (delayed plating), they were trypsinized, and 800 cells were seeded per plate. After 14 days, the colonies formed were stained by 6% glutaraldehyde and 0.5% crystal violet. The stained colonies were manually counted. A colony was defined to consist of at least 50 cells, and an inverted fluorescence microscope (IX73, Olympus, Tokyo, Japan) was used for assessment of colony size. The experiment was carried out in triplicate and three independent experiments.

### 2.6. Histone H2A.X Phosphorylation Assay

PC3 cells were divided into four groups: the control group (H_2_O), the group treated with SCNdU (50 μM, 24 h of incubation), the irradiated (2 Gy, Cellrad X-ray cabinet, Faxitron X-ray Corporation) group, and the combination group (exposed to 50 μM SCNdU and 2 Gy of radiation). After irradiation, the cells were incubated for 1 or 24 h. Then, the cells were dissociated with accutase solution, fixed, and permeabilized. Next, the cells were stained with labelled antibody and analyzed by a Guava easyCyte flow cytometer. All manipulations (fixation, permeabilization, and staining) were performed according to the manufacturer’s protocol (FlowCellect Histone H2A.X Phosphorylation Assay Kit, Merck, Darmstadt, Germany). Unirradiated controls were used to set the threshold gating to determine the percentage of γH2A.X positive cells. In addition, the positive controls (cells irradiated with high dose, immediately after irradiation) were routinely included for comparison and setting the threshold. The gating strategy has been shown in Figure S3 in the Supplementary Material. The experiment was carried out in triplicate.

### 2.7. Immunofluorescence Microscopy

Cells were divided into four groups as described above. They were grown on polymer coverslips (µ-slide, Ibidi, Gräfelfing, Germany), fixed with 4% paraformaldehyde in PBS for 10 min (1 h after irradiation), and permeabilized with 0.2% Triton X-100 in PBS for 5 min. Next, they were washed in PBS, incubated with the rabbit anti-gamma H2A.X (phospho S139) antibody (1:4000, Abcam, Cambridge, UK), followed by anti-rabbit AlexaFluor 546-conjugated IgG (1:1000, Thermofisher, Waltham, MA, USA), and analyzed using a Leica TCS SP8X confocal laser scanning microscope with a 63× oil immersion lens (Leica Microsystems, Wetzlar, Germany). Nuclear staining was performed using 4′,6-diamidino-2-phenylindole (DAPI). γH2A.X foci were quantified as foci per nucleus. γH2A.X foci were scored using ImageJ software in at least 80–100 cells.

### 2.8. Cell Death Analysis

Cells were divided into four groups as described in Section 2.4. Twenty-four hours after exposure to radiation, the cells were dissociated with accutase solution, stained with MitoSense and 7-AAD, and analyzed using a Guava easyCyte flow cytometer according to the manufacturer’s protocol (FlowCellect MitoDamage Kit, Merck).

### 2.9. Statistical Analysis

Mean values were statistically compared using one way ANOVA with Dunnett’s or Tukey’s multiple comparison test. The data are represented as means and the standard deviations (SD) of the mean from at least three independent experiments. Data were statistically analyzed using GraphPad Prism 7 software. Statistical significance: * *p* < 0.05, ** *p* < 0.01, *** *p* < 0.001, **** *p* < 0.0001.

## 3. Results

### 3.1. Cytotoxicity of SCNdU

**Figure 1:**
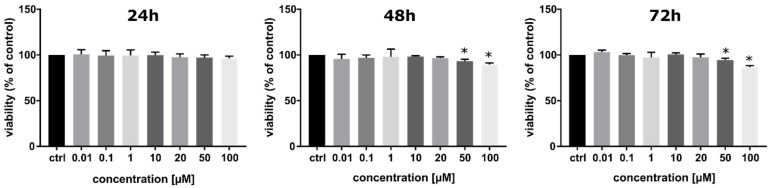
Viability of PC3 cells after 24, 48, and 72 h of incubation with SCNdU in the concentration range from 0.01 µM to 100 µM. The results are shown as the mean ± SD of three independent experiments performed in triplicate. * A statistically significant difference (*p* < 0.05) is present between the treated samples and control (an untreated sample).

Using SCNdU as a radiosensitizer requires that it should have low cytotoxicity without exposure to ionizing radiation. For this reason, the impact of SCNdU treatment on PC3 cells viability was determined by the MTT test [17]. It was shown that the reduction of viability generated by SCNdU compared to the viability of cells in the control variant (untreated cells) is statistically significant (*p* < 0.05) only in concentrations ≥50 µM and for 48 and 72 h of incubation (Figure 1). For 48 h incubation, treatment with 50 µM and 100 µM SCNdU reduces cell viability to 93.2 ± 2% and 89.6 ± 1.4%, respectively. The viability of PC3 cells pretreated with 50 µM and 100 µM SCNdU for 72 h was equal to 94.4 ± 1.8% and 86.7 ± 1.5%, respectively. In other cases, a statistically significant decrease in viability of PC3 cells was not observed.

### 3.2. Clonogenic Survival of PC3 Cells Treated with SCNdU

**Figure 2:**
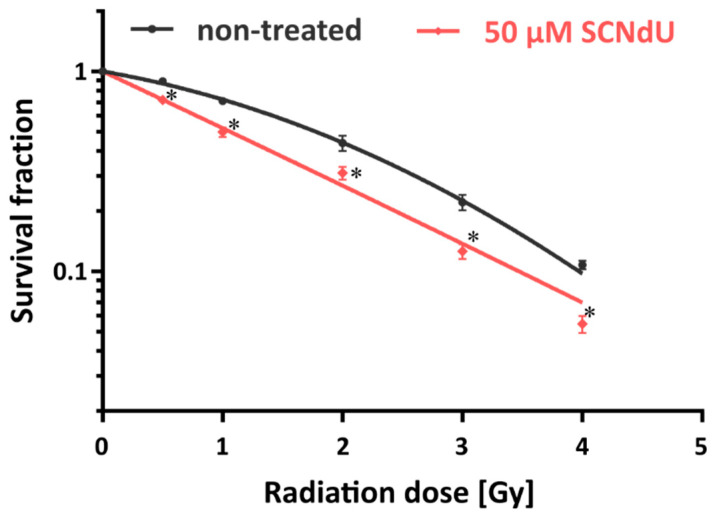
Dose–response curves of PC3 cells treated with 50 µM SCNdU or without SCNdU. Experiments were performed in three independent experiments in triplicate, and the results are expressed as mean ± SD. * significant difference compared with the untreated variants, *p* < 0.05.

To determine the impact of SCNdU on the survival and colony formation ability of prostate cancer cells exposed to ionizing radiation, a clonogenic assay [18] was performed. Figure 2 shows that the tested compound significantly influences the proliferation ability of the X-irradiated tumor cells. Namely, a significant reduction of dividing cells, caused by exposure to radiation as well as incubation with SCNdU, is depicted by Figure 2. The treatment of the PC3 cells by SCNdU brings about a reduction of the survival fraction from 71.2 ± 2.1% to 49.7 ± 2.7% for a 1 Gy dose and from 43.9 ± 3.9% to 30.9 ± 2.3% for a dose of 2 Gy. The dose enhancement factor (ID50(−treatment)/ID50(+treatment)) [19], where ID50 means radiation dose causing 50% growth inhibition), representing the radiosensitizing effect of SCNdU, is equal to 1.67. These results suggest that SCNdU significantly sensitizes prostate cancer cells to ionizing radiation.

### 3.3. Impact of SCNdU on PC3 Cell Cycle

**Figure 3:**
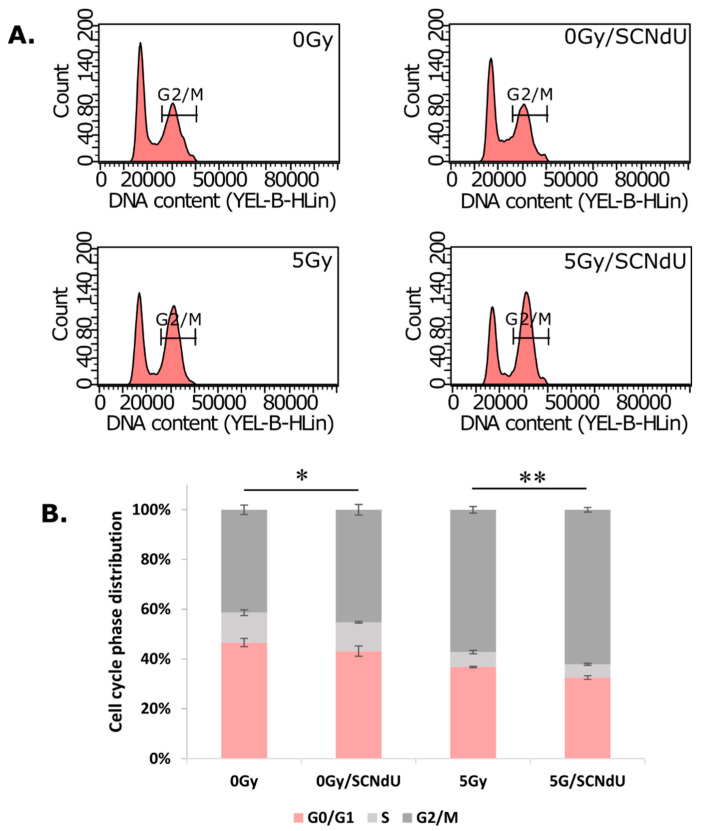
Effect of SCNdU on PC3 cell cycle distribution. (**A**) Cells were pretreated with SCNdU for 24 h before exposure to 0 Gy or 5 Gy radiation, and the fraction of cells in each phase of the cell cycle was analyzed by flow cytometry. (**B**) Quantitative analysis of cell cycle distribution. The results are expressed as mean ± SD. Statistical notations for differences in cell populations in G2/M between indicated groups * *p* < 0.05, ** *p* < 0.01.

To clarify the mechanism by which SCNdU enhances cancer cell sensitivity to radiation, we examined the effects of combined treatment on cell cycle regulation by flow cytometry. Treatment with SCNdU and radiation decreases the fraction of cells in the G0/G1 phase and increases their fraction in the G2/M phase when compared to the untreated control cells (Figure 3). Indeed, the G2/M fraction reached 57.18 ± 1.3% and 62.12 ± 0.94% for the PC3 cells irradiated with 5 Gy and irradiated together with the SCNdU treatment, respectively. This fraction was equal to only 41.21 ± 1.90% for the control cells and 45.14 ± 2.13% for treatment with SCNdU at the concentration of 50 μM. The impact of SCNdU treatment (without irradiation) in a concentration range of 10–100 μM on cell cycle distribution is presented in Figure S2 in the Supplementary Materials. A statistically significant difference between SCNdU-treated cells in G2/M phase and the control (an untreated culture) is present in case of 50 µM and 100 µM SCNdU concentrations. On the other hand, the cell population in the G0/G1 phase decreased from 46.38 ± 1.66% (control) to 43.00 ± 2.07% (SCNdU treatment) and from 36.72 ± 0.28% (5 Gy) to 32.50 ± 0.74% (a combined use of the SCNdU treatment and 5 Gy of X-ray).

### 3.4. Double Strand Break Formation

**Figure 4:**
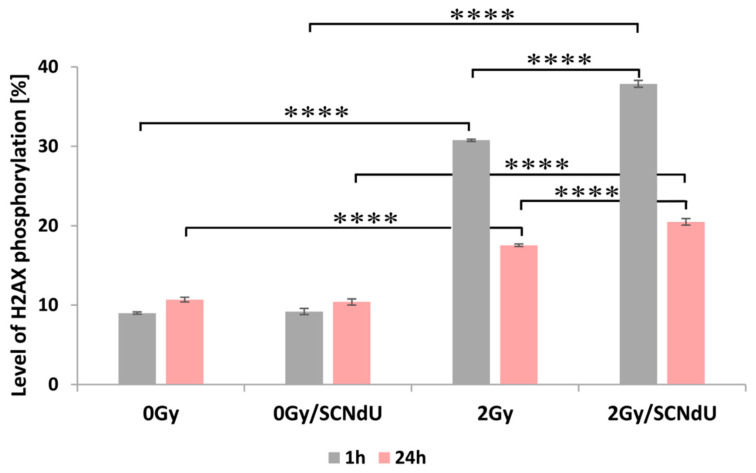
Analysis of H2A.X histone phosphorylation by flow cytometry. The level of γH2A.X was measured 1 h and 24 h after irradiation. The results are shown as the mean ± SD of three independent experiments performed in triplicate. **** Statistically significant difference between indicated groups is present at *p* < 0.0001. The level of H2A.X phosphorylation means the percentage size of population for which cell fluorescence exceeds a gating limit for H2A.X label.

One of the most common types of DNA damage related to radiosensitization is DNA double-strand breaks (DSBs). A molecular marker of this damage is phosphorylation of the H2A.X histone [20]. The cytometric analysis of this process is shown in Figure 4, Figure S3 and Table S1 in the Supplementary Materials. The presented results show that the treatment of cells by SCNdU alone, without exposure to X-rays, did not increase the population of positive cells (for the definition of positive cells population, see caption of Figure 4 and the gating strategy in Figure S3 in Supplementary Material; high fluorescence signal meaning high level of histone phosphorylation related to the appearance of DSBs). However, exposing them to SCNdU followed by irradiation with a dose of 2 Gy increases the level of histone H2A.X phosphorylation from 30.77 ± 0.14% (for the untreated, irradiated culture) to 37.87 ± 0.43%. After 24 h from irradiation (in this time potentially lethal damage repair is complete [21]), the population of positive cells in the irradiated culture assumes 17.54 ± 0.16%, while it increases to 20.49 ± 0.41% for the cells irradiated and pretreated with SCNdU.

**Figure 5:**
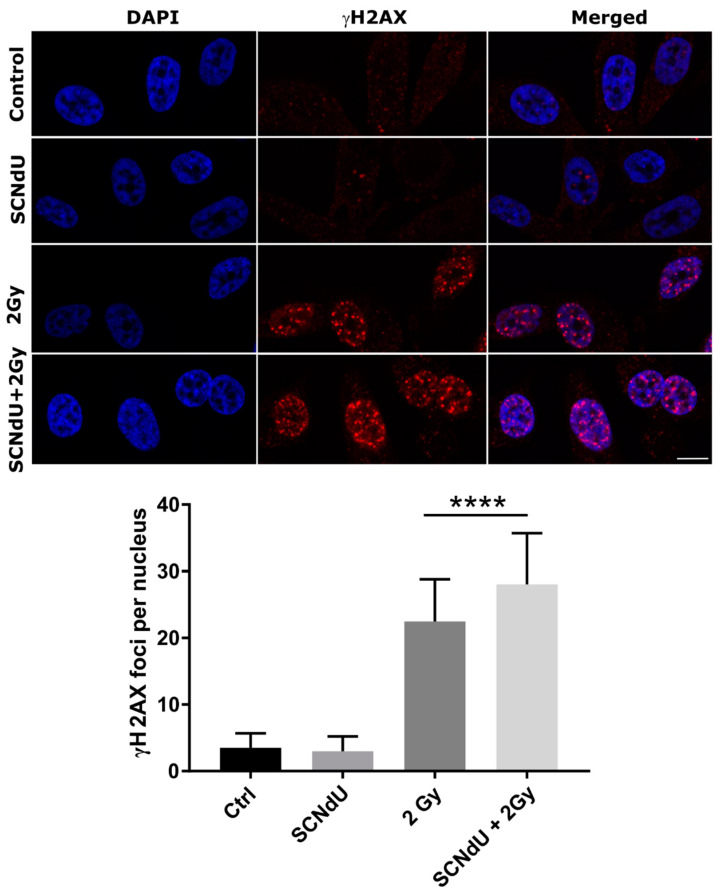
The representative images of γH2A.X foci immunofluorescence staining in PC3 cells (control cells, treated with 50 µM SCNdU, irradiated with 2 Gy, treated with 50 µM SCNdU and irradiated with 2 Gy). Cells were harvested 1 h following IR. Slides were counterstained with DAPI to visualize nuclei (left panels). γH2A.X foci were quantified as number of foci per nucleus. The results are shown as the mean ± SD. **** Statistically significant difference at *p* < 0.0001. Specimens were analyzed using confocal laser scanning microscope (Leica SP8X) with a 63× oil immersion lens (Scale bar −10 µm).

Among different assays for γH2A.X detection, immunofluorescent microscopic γH2A.X foci determination is still considered to be the most sensitive approach. For this reason, the number of foci per nucleus was additionally examined. Figure 5 shows the immunofluorescence staining of (i) irradiated, (ii) SCNdU-treated, (iii) SCNdU-treated and irradiated cells, as well as (iv) the control culture (untreated and unirradiated cells). As shown in Figure 5, SCNdU significantly increased the number of γH2A.X foci per nucleus at 24 h incubation following 2 Gy radiation compared to that of radiation alone (28.1 ± 7.7 compared to 22.5 ± 6.3, *p* < 0.05). These results suggested that the combination of SCNdU and radiation increased DNA damage.

### 3.5. Analysis of Cell Death

**Figure 6:**
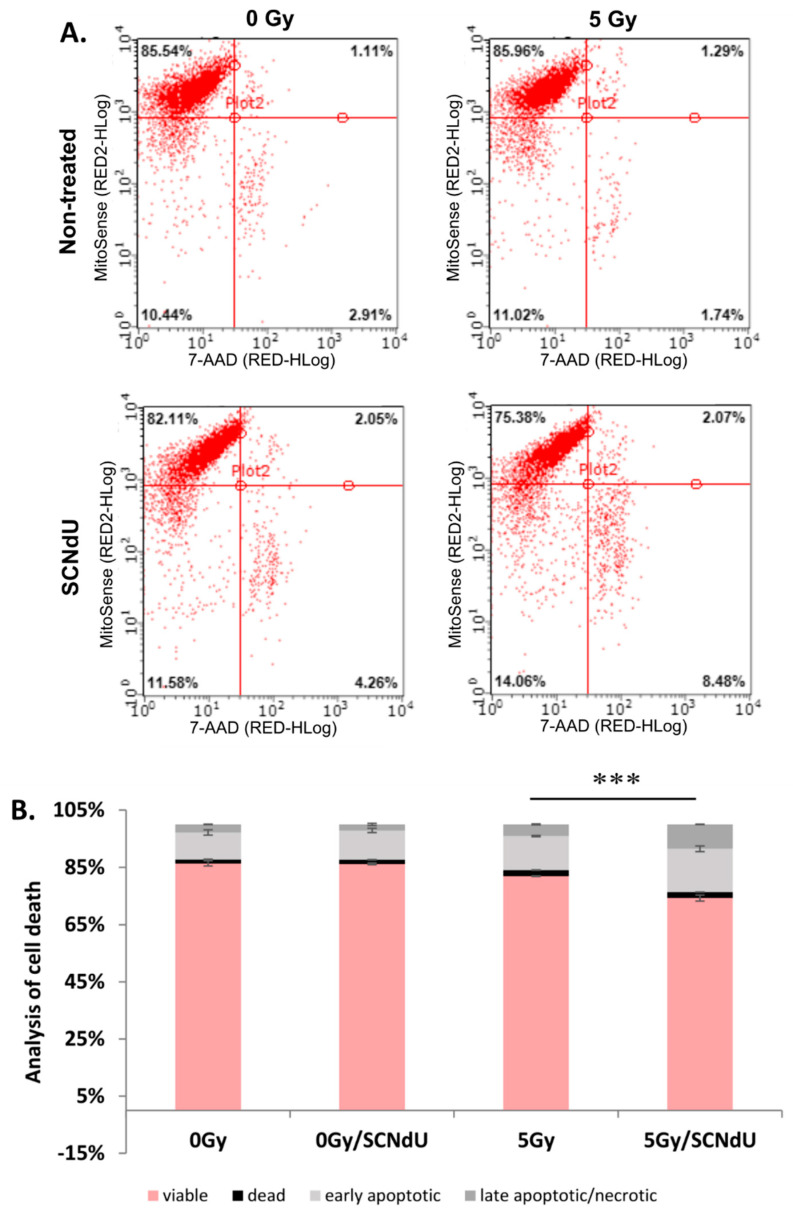
Cytometric analysis of cell death. (**A**) Dot plots provide comparison of MitoSense Red vs. 7AAD. Q1 upper left quadrant, viable (MitoSense Red+ 7-AAD−), Q2 lower left quadrant, early apoptotic (MitoSense Red− 7-AAD−), Q3 lower right quadrant, late apoptotic/necrotic (MitoSense Red− 7-AAD+), and Q4 upper right quadrant, dead cells (MitoSense Red+ 7-AAD+). (**B**) Quantitative analysis of cell death (measurement of viable, dead, early apoptotic, and late apoptotic/necrotic cell populations). The results are shown as the mean ± SD of three independent experiments performed in triplicate. *** Statistically significant difference between early apoptotic cell populations is present at *p* < 0.001.

To determine cell death following combined treatment with SCNdU and radiation, the cells were analyzed by MitoSense/7-Aminoactinomycin-D (7AAD) staining and flow cytometry. MitoSense Red is a dye that accumulates in the mitochondria and indicates changes in mitochondrial membrane potential. The measurement of mitochondrial membrane potential appears as a crucial indicator of mitochondrial dysfunction, and it has been considered to be an early hallmark of apoptosis. The 7-AAD dye monitors the cell membrane permeability changes typically observed later in apoptosis as well as necrotic cells. Therefore, labeling by the MitoSense Red dye that has accumulated in healthy mitochondria and 7-AAD labeling the dead cells allow identifying live, apoptotic, and dead cells, respectively (Figure 6). Pretreatment of cells with SCNdU does not affect the population of viable, early apoptotic, late apoptotic, and dead cells compared to the control variant (non-treated with SCNdU or radiation). However, simultaneous action of radiation and SCNdU decreased the viable cell population from 81.98 ± 0.13% to 74.32 ± 1.07% and increased the early apoptotic cell population from 11.80 ± 0.22% to 15.05 ± 0.99%, in comparison to the cells exposed only to radiation. Also, the level of late apoptotic/necrotic cells increased due to the combined use of radiation and the tested compound (4.10 ± 0.17% in the case of irradiated cells and 8.54 ± 0.06% in the case of cells irradiated and pretreated with SCNdU).

## 4. Discussion

RT is a common modality of cancer treatment. Efforts to improve the therapeutic ratio of radiation by pharmacologic means have led to important advances in the care of cancer patients. One of the most promising approaches is to combine radiosensitizing agents with RT to improve tumor response rates. This facilitates the use of RT at lower doses with higher efficacies. It is expected that agents modifying tumor response to radiation therapy act by altering one or more of the “5 Rs of radiobiology”: inherent cellular radiosensitivity, repair, reassortment, repopulation, and reoxygenation [22]. Indeed, the action of one of the best known radiosensitizers—nimorazole—can be described in this manner. This agent acts as a selective tumor radiosensitizer by preferentially targeting hypoxic tumor cells, which are otherwise relatively radioresistant. Nimorazole significantly improves the effect of radiotherapeutic management (by 16% compared to radiation therapy alone) in patients with cancer of the supraglottic larynx and pharynx, and it can be given without major side effects and excess toxicity [23]. Nimorazole is routinely used only in Denmark [24].

Radiation therapies are commonly used in the management of prostate cancer since it is highly curable with radiation [25,26]. RT treatment, in this case, is also associated with an acceptable toxicity [27]. Nevertheless, the possible advantages of radiosensitization might allow dose de-escalation with the consequent benefits in terms of toxicity reduction. For this reason, it seems reasonable to develop novel strategies to improve the control of prostate cancers, including proposing and studying novel radiosensitizers.

In this paper, we study the radiosensitizing properties of SCNdU towards PC3 cells. This compound is a representative of the modified nucleosides which are considered to be very promising candidates for effective radiosensitizers [7,28,29]. The selectivity of such compounds results from cancer cells’ ability for rapid growth and uncontrolled division. However, if the nucleosides are appropriately structurally modified, they can additionally engage hypoxia as a second selectivity criterion. Such modification should rely on the introduction of substituents that increase nucleosides’ sensitivity to degradation induced by solvated electrons, which are the second major product of water radiolysis under hypoxic conditions [7]. The radiation chemical studies of electron addition to SCNdU establish this analog as a potential radiosensitizer causing DNA damage [15,16]. Here, for the first time, we confirm its radiosensitizing action towards prostate cancer cells. We demonstrate that the derivative studied is marginally cytotoxic without exposure to ionizing radiation. A statistically significant decrease in viability of PC3 cells was observed only in case of treatment with SCNdU at the relatively high concentrations of 50 µM and 100 µM SCNdU and for the contact times of 48 and 72 h. The situation is completely different in case of radiation exposure—SCNdU significantly decreases the proliferation ability of the X-irradiated prostate cancer cells. The dose enhancement factor (expressed as the ratio of doses reducing survival to 50%,) representing the radiosensitizing effect of SCNdU, is equal to 1.69. To get deeper insight into the mechanism of SCNdU-mediated radiosensitization, we checked the impact of this analog on the PC3 cell cycle. These results indicate that simultaneous exposure of cells to SCNdU and radiation causes acceleration through G1/S and arrest in the G2/M phase. Since cells are most sensitive to the effects of radiation in the latter phase and most resistant in the G0/G1 phase, a potential mechanism by which SCNdU enhances the effects of ionizing radiation seems to be by regulating cell cycle progression. Modified nucleosides are designed to primarily cause DNA damage. For this reason, the impact of SCNdU treatment on double strand break formation after irradiation has been studied. It was found that exposing PC3 cells to SCNdU followed by irradiation with a dose of 2 Gy significantly increases the level of histone H2A.X phosphorylation correlating with the amount of DSBs. The ultimate desired response of a cell to ionizing radiation is cell death. Therefore, cell death analysis was performed following combined treatment with SCNdU and radiation. The pretreatment of prostate cancer cells with SCNdU does not affect the population of viable, early apoptotic, late apoptotic, and dead cells. This is in contrast to the simultaneous action of radiation and SCNdU. The combined use of both agents caused an increase in the level of the early apoptotic and late apoptotic/necrotic cells.

Our results encourage further investigation on radiosensitization by SCNdU. First of all, we are going to investigate the influence of hypoxia on the effectiveness of radiosensitization (the expected increase in radiosensitization of cancer cells under hypoxia associated with the greater electron production) and study in more detail the mechanism of radiosensitization. Other issues include determining the efficacy in vivo, biodistribution, and toxicity. In particular, reaching a therapeutic level of SCNdU in cancer cells may be a limitation for use in humans. This was the case with one of the most effective radiosensitizing modified nucleosides—5-bromo-2′-deoxyuridine [30,31,32]. It has been proven that more than 90% of this halogenated pyrimidine administered intravenously is deactivated (by dehalogenation) in the liver within one hour [33].

## 5. Conclusions

In this paper, we verify the radiosensitizing properties of SCNdU at the cellular level. SCNdU belongs to the group of modified nucleosides which undergo dissociative electron attachment induced by solvated electrons (one of the major products of water radiolysis under the hypoxic conditions that are characteristic of solid tumors) that are negligibly reactive towards native DNA. The results of the executed analyses demonstrate that SCNdU possesses radiosensitizing properties at the cellular level, and thus, complements previous model studies on this derivative which suggested that SCNdU is a radiosensitizer. This analogue increases the sensitivity of PC3 on ionizing radiation. This is, at least partially, related to an increase in the number of DNA double-strand breaks. These results demonstrate that the radiosensitizing effect of SCNdU treatment is also associated with cell cycle regulation and apoptosis induction. What is important is that the derivative is marginally cytotoxic without exposure to ionizing radiation. In summary, our results encourage a further important step, i.e., in vivo studies that are a necessary precondition of clinical trials on new radiosensitizers—compounds belonging to a class of drugs which is still underrepresented in anticancer treatment.

## Data Availability

Research data are stored in an institutional repository and will be shared upon a request to the corresponding author.

## Supplementary Materials

The following supporting information can be downloaded at: https://www.mdpi.com/article/10.3390/cancers14082035/s1, Figure S1: Purity of SCNdU, Figure S2: Impact of SCNdU on cell cycle, Figure S3 and Table S1: Cytometric analysis of histone H2A.X phosphorylation.
