# Supplementary material for: Radiosensitization of PC3 Prostate Cancer Cells by 5-Thiocyanato-2′-deoxyuridine
Source: Cancers (Basel). 2022 Apr 18;14(8):2035. doi: 10.3390/cancers14082035 (PMC9025292; doi:10.3390/cancers14082035)
Supplement: Supplementary file 1 [file cancers-14-02035-s001.zip › cancers-1694280-supplementary.pdf]

Supplementary Materials

Radiosensitization of PC3 prostate cancer cells by 5-thiocyanato-2’-deoxyuridine

Magdalena Zdrowowicz[1]*, Magdalena Datta[1], Michał Rychłowski[2], Janusz Rak[1]

1 Laboratory of Biological Sensitizers, Faculty of Chemistry, University of Gdańsk, Wita Stwosza 63, 80-308 Gdańsk, Poland

2 Laboratory of Virus Molecular Biology, Intercollegiate Faculty of Biotechnology, University of Gdańsk—Medical University of Gdańsk, Abrahama 58, 80-307 Gdańsk, Poland

* Correspondence: magdalena.zdrowowicz@ug.edu.pl

Purity of SCNdU

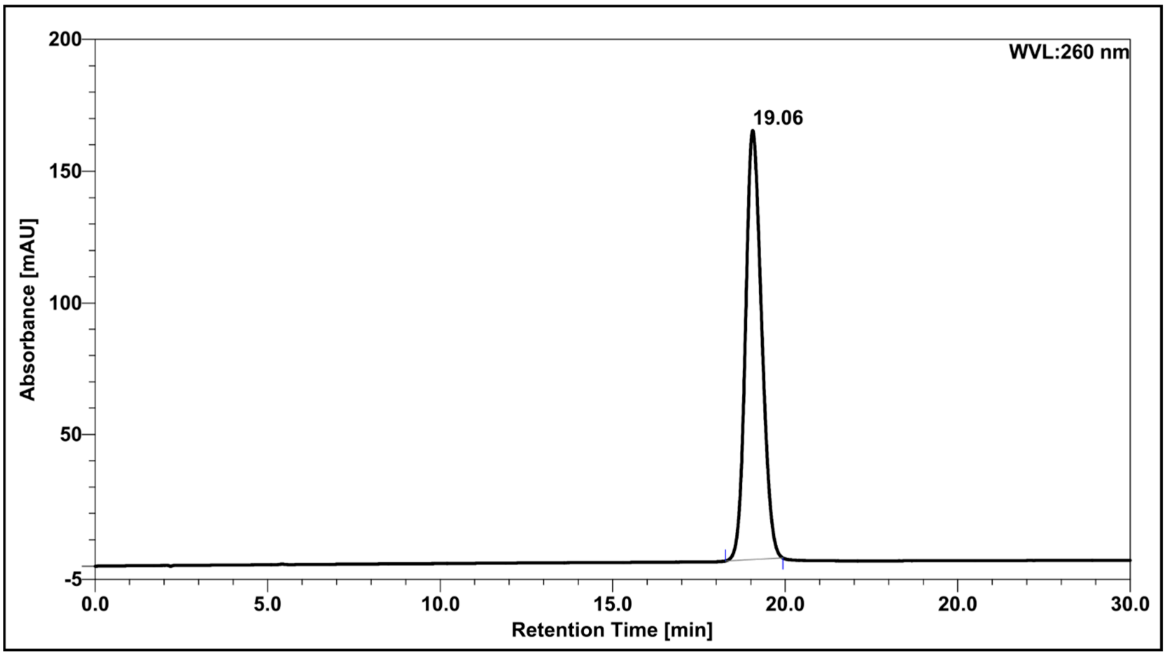

Figure S1. Chromatogram of SCNdU solution. HPLC conditions: reversed-phase HPLC method; C18 column (Wakopak Handy ODS, 4.6 · 150 mm, 5 µm in particle size and 100 Å in pore size); elution with 0.1% HCOOH; flow rate 1 mL · min−1; DionexUltiMate 3000 System with a Diode Array Detector set at 260 nm.

Impact of SCNdU on cell cycle

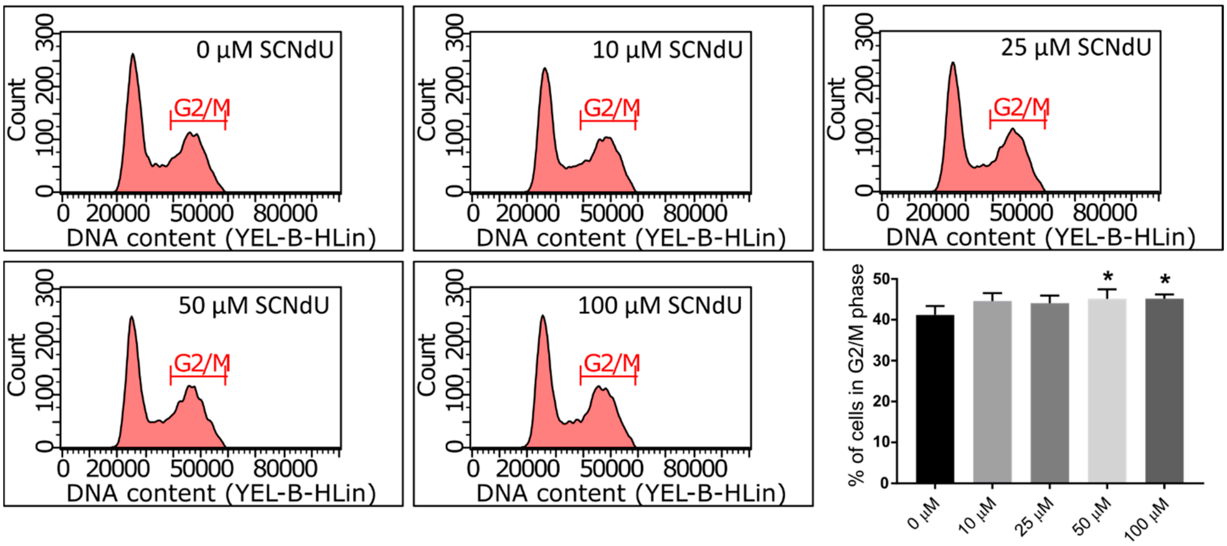

Figure S2. Impact of SCNdU (0-100 µM), without irradiation, on cell cycle. Quantitative bar graph presents the percent of cells in G2/M phase. Values are shown as the means ± SD of three independent experiments. *Statistically significant difference ($p < 0.05$) is present between the treated samples and control (untreated sample) (ANOVA, with Dunnett's multiple comparison test).

Cytometric analysis of histone H2A.X phosphorylation

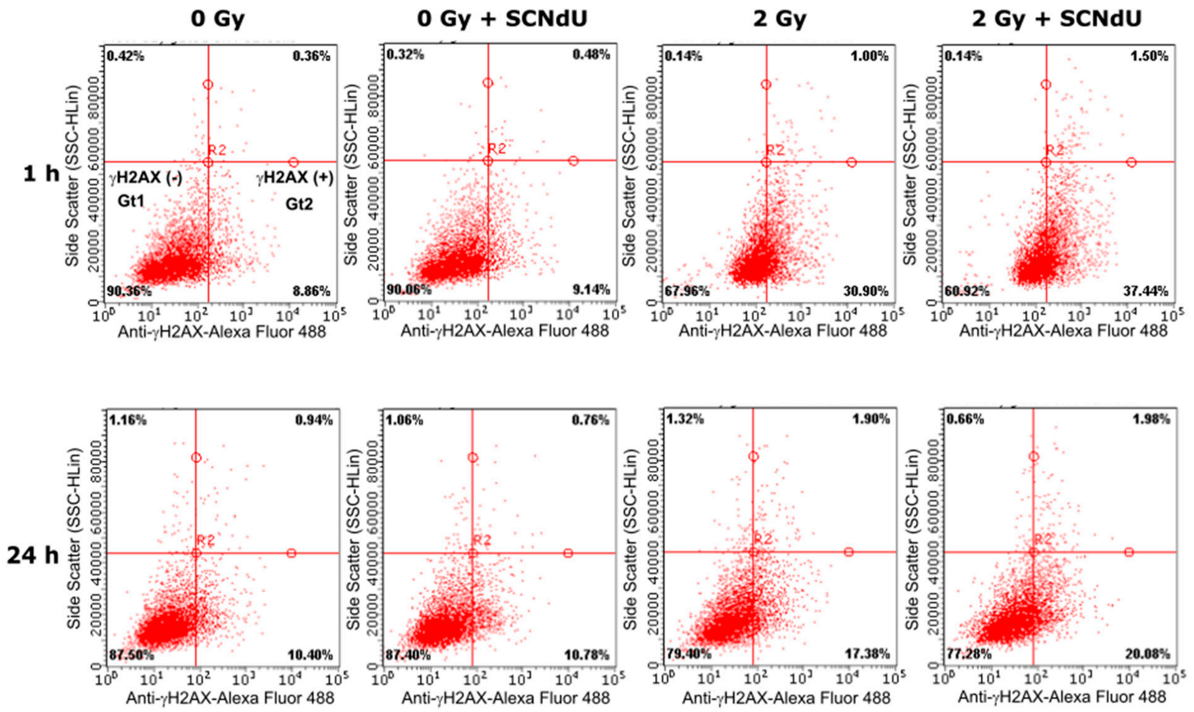

Figure S3. Cytometric analysis of histone H2A.X phosphorylation. Viable cells are gated on the scatter plot FSC (forward scatter) versus SSC (side scatter) for exclusion of cell debris and aggregates. On the bivariate dot plot SSC versus 488 nm laser area (Alexa Fluor 488-A), γ-H2AX-positive cells depicted in the right quadrants and γ-H2AX-negative cells depicted in the left quadrants are gated in control cell population. The same gating is applied for SCNdU treated and irradiated cells.

Table S1. Quantitative, cytometric analysis of histone H2A.X phosphorylation.

Time (from irradiation)	Population of positive cells (histone H2A.X phosphorylation) [%]			
	0 Gy	0 Gy + SCNdU	2 Gy	2 Gy +SCNdU
1 h	9.00±0.11	9.19±0.39	30.77±0.14	37.87±0.43
24 h	10.70±0.30	10.40±0.39	17.54±0.16	20.49±0.41
